# Tungsten-Based Hybrid Composite Shield for Medical Radioisotope Defense

**DOI:** 10.3390/ma15041338

**Published:** 2022-02-11

**Authors:** Seon-Chil Kim

**Affiliations:** Department of Biomedical Engineering, School of Medicine, Keimyung University, 1095 Dalgubeol-daero, Daegu 42601, Korea; chil@kmu.ac.kr; Tel.: +82-10-4803-7773

**Keywords:** hybrid fabric, shielding fiber, radiation shield, medical radiation, nuclear medicine test

## Abstract

The shielding performance of shielding clothing is typically improved by increasing the shielding material content, but this lowers the tensile strength of the material. The weight and wearability of the shielding suit are also adversely affected. Important considerations when developing shielding fabric are thickness and flexibility to allow the wearer sufficient mobility. Insufficient thickness lowers the shielding performance, whereas excessive thickness decreases the flexibility of the garment. This study aimed to develop a composite shield that reproduces the shielding performance and meets the flexibility of the process technology. The new shield was manufactured by combining two layers: the shielding fabric fabricated from tungsten wire and a shielding sheet produced by mixing a polymer (PDMS) with tungsten powder. These two shields were bonded to develop a double hybrid composite. Compared with the existing shielding sheet (produced from lead equivalent of 0.55 mmPb), the shielding performance of the hybrid composite shield improved by approximately 17% on average and the tensile strength was 53% higher. The hybrid composite shield has a thickness of 1.35 ± 0.02 mm and delivers the same shielding performance as the lead equivalent. The new hybrid composite shield offers higher wearer mobility while shielding against radiation exposure in medical institutions.

## 1. Introduction

Radiation is used in medical institutions for the diagnosis and treatment of a range of conditions. In particular, the X-rays used for diagnostic purposes have a relatively low energy in the range 40–150 kVp and are mainly used in general imaging laboratories. In contrast, high-energy gamma rays of 140 keV, 364 keV, and 511 keV are mainly used for nuclear medicine imaging tests [1]. Nuclear medicine is a field that diagnoses and treats the anatomy, physiology, and biochemical states of the body using specific properties of radioactive and stable nuclides [2,3,4]. The radiation emitted from isotopes used in nuclear medicine has unique characteristics, and medical personnel cannot artificially control its properties.

Preventive measures for radiation exposure in medical institutions are of critical importance. When exposed to radiation, the greatest effect on the human body is cell tissue damage. The body may repair minimal damage, but if the damage is more severe, it may not recover. This can lead to chromosome damage and potentially genetic modification [5,6,7]. Specifically, as gamma rays are high-energy photons with short wavelengths and high frequencies, and as they are emitted in high volume, they can easily penetrate the human body; thus, active defense is vital.

The radiation used in nuclear medicine applications has high energy and transmittance, and because of its lack of direction, it is difficult to shield against. As a result, workers may have a high risk of exposure in medical environments [8]. Therefore, radiation shielding for nuclear medicine practitioners is essential. In general, radiation is shielded by being absorbed by the atoms constituting the shield due to high-density materials and heavy-atoms [9]. Lead is the most commonly used radiation shielding material in medical institutions [10,11], as it has excellent radiation shielding ability owing to its high atomic number and density. A lead apron is a shield that can be worn as clothing, which can effectively protect radiation workers from exposure.

However, lead is toxic and harmful to both the human body and the environment [12]. In addition, lead-based clothing intended for shielding weighs 3.15–3.45 kg based on a lead equivalent of 0.25 mmPb, which limits the mobility of medical personnel and places a physical burden on users. Accordingly, a need has arisen for a new shielding material to replace the lead currently used to shield personnel working with nuclear medicine from gamma rays.

Eco-friendly shielding materials that can replace lead include tungsten, bismuth oxide (Bi_2_O_3_, 8.9 g/cm^3^), barium sulfate (BaSO_4_, 4.5 g/cm^3^), and boron (B, 2.08 g/cm^3^) [13]. Radiation shielding materials that are considered to be eco-friendly are materials that are harmless to the human body and pose no risk of contamination during manufacturing and disposal [14].

Of these, tungsten has an atomic number of 74, which is similar to that of lead (82), has a density of 19.25 g/cm^3^, which is considered high density, and exhibits the most similar shielding effect to lead [15]. Additionally, radiation shields manufactured using tungsten are lighter than lead, meaning protective clothing is less burdensome.

The material from which the radiation-shielding suit is manufactured is conventionally produced by mixing the shielding material in the form of a powder with a polymer and is mainly produced in the form of a sheet or film [16]. In addition, in certain cases, a fibrous tissue such as a non-woven fabric is attached to the surface of the shielding sheet to improve flexibility or prevent external contamination. These methods also serve to supplement the low sheet tensile strength of 24.2–60 MPa [17].

The core of the shielding sheet manufacturing process is the dispersibility of the shielding material, in this case, tungsten particles. The dispersibility affects the maintenance of uniform shielding performance. To improve the dispersibility of the particles, nano-sized particles or increasing the mixability using polymers such as chromium (CR), ethylene vinyl acetate (EVA), and thermoplastic polyurethane (TPU) may be used [18]. However, shielding against high-energy radiation such as gamma rays requires a shield of 0.55 mmPb, and because the shield needs to comply with the minimum thickness and flexibility requirements, it is necessary to find ways to modify the sheet manufacturing process with the aim of reducing the thickness [19].

This study led to the development of a hybrid composite shield manufactured by using shielding fiber comprising tungsten wire as the outer shell of the shielding sheet. The purpose of the work was to improve the shielding performance and the tensile strength of the existing sheet. In practice, this involved solving the problem of the tensile strength being lowered by increasing the shielding material content when manufacturing the shielding sheet, the reason for supplementing it with fibers with shielding properties.

After bonding a shielding sheet made by mixing a flexible tungsten wire-based shielding fiber with tungsten nanopowder and the polymer polydimethyl siloxane (PDMS), a tungsten-based hybrid composite shield was manufactured. This shield is expected to produce superior shielding performance compared to conventional nonwoven fabric.

The shielding performance and tensile strength of the manufactured shield were compared and analyzed to evaluate the suitability of the process. The shielding fiber and shielding sheet manufactured using the tungsten wire were bonded, and the developed shield processing method is presented, along with a technological plan for commercialization and mass production. In addition, the proposed technology is intended to ensure that medical personnel working in medical institutions are allowed the necessary mobility by providing them with shielding clothing with sufficient thickness and flexibility.

## 2. Materials and Methods

**Figure 1:**
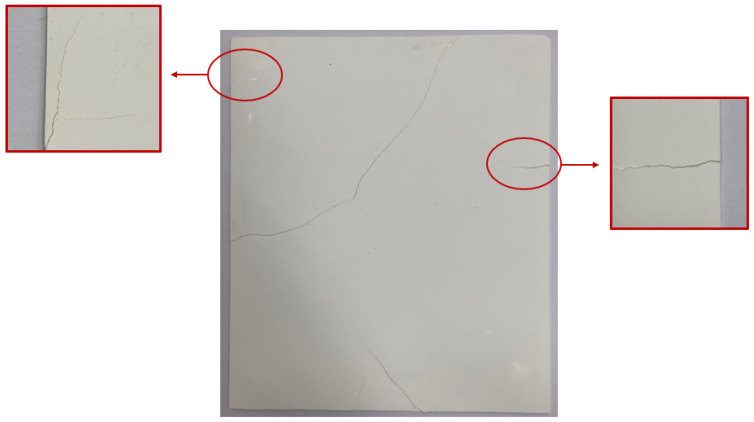
Cracks due to the aging of the shielding sheet lower the shield performance.

Existing shielding sheets were manufactured through thermocompression coating technology by mixing a polymer with a shielding material such as tungsten [20]. In such a shielding sheet, cracks may occur in the bent portion, as displayed in Figure 1, owing to low durability. In this study, to reduce the surface cutting phenomenon caused by such physical impact, the shielding sheet was bonded to the surface of the fiber woven with tungsten wire and a shielding yarn.

According to the Beer–Lambert law,

(1) $$I=I_{0}e^{-\left(\frac{\mu }{\rho }\right)t},$$

where $I_{0}$ and I are the incident intensity of an initial photon without attenuation and the intensity attenuates as it passes through the shielding sheet, respectively, the intensity of the X-ray beam energy is attenuated within the shielding material using the same principle as [21]. Additionally, $\frac{\mu }{\rho }$ (g/cm^2^) is the mass attenuation coefficient, and t is the thickness of the shielding sheet corresponding to the mass per unit area (g/cm^2^). The mass thickness of the shielding sheet varies according to the polymer and tungsten nano-powder content [22]. Subsequently, the mass attenuation coefficient of a mixture or multi-element compound can be determined [23]:

(2) $$\frac{\mu }{\rho }=\underset{i}{\sum }W_{i}{\left(\frac{\mu }{\rho }\right)}_{i},$$

where $W_{i}$ is the mass percentage (wt%) of each element contained in the mixture. Therefore, the thickness of the shielding sheet plays the most important role in the shielding performance. In addition, the shielding performance of the sheet is determined by the mass percentage of the polymer and the shielding material. Consequently, to increase the mass per unit area of the shielding material, the content of the shielding material must be increased. However, as increasing the shielding material content decreases polymer content, the tensile strength of the sheet decreases due to the weakening of the bonding force between the particles.

**Figure 2:**
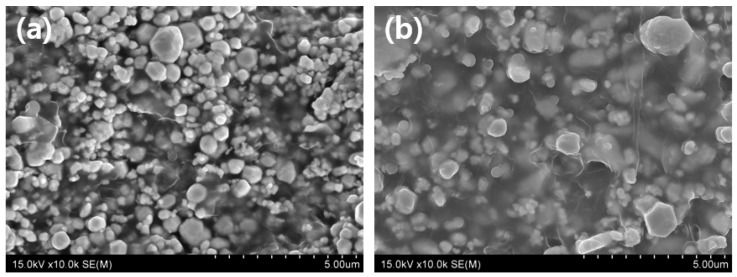
Cross-section of the internal structure of the shielding sheet based on the amount of polymer, with (**a**) containing 80 wt% tungsten and (**b**) containing 60 wt% of tungsten.

Figure 2 shows a cross-sectional comparison of the shielding sheet according to the polymer ratio. Figure 2b shows that the shielding sheet (contains tungsten 60 wt%) has a polymer ratio approximately twice that of Figure 2a he shielding sheet (contains tungsten 80 wt%). It can be seen that the shielding material in Figure 2a is not sufficiently dispersed, and pinholes appear in various locations [24].

In this study, to alleviate the reduction in tensile strength of the sheet, a composite yarn covered with a polyethylene terephthalate (PET) yarn was manufactured on a tungsten wire (Beijing Tianlong tungsten Technology Co. Ltd., Beijing, China) with a purity of 98% and a diameter of 30 μm [25]. The PET yarn was made using barium sulfate and bismuth oxide to increase the shielding performance. Each master batch including these two materials was kneaded with natural PET at 290 °C, and then two types of yarn each containing 5 wt% were produced by melt spinning [26]. Table 1 shows the weave characteristics of the fibers used as the outer skin of the shielding sheet.

The tungsten powder (purity 99.9%) used for manufacturing the shielding sheet was subjected to ultrasonic grinding at least five times to a size of 10 μm or less using a Fisher sub-sieve sizer, and then dried in an oven at 60 °C for 24 h. Polydimethyl siloxane (PDMS) was used as a polymer, as it is a material with excellent thermal stability. As the intermolecular force is weak, it also has excellent compatibility with mixed materials [27].

As a solvent, N-dimethylformamide (DMF, 99.5%) was used. For solution-cast, first a certain proportion of PDMS and DMF were completely dissolved and left for at least 12 h to remove internal air bubbles. Additionally, in this state, tungsten powder was added and stirred for 10 to 15 min at a speed of 3000 rpm, dispersing the tungsten particles. In this process, the content of tungsten powder was made up to 90 wt% with the PDMS.

The additives used in the sheet manufacturing process include colorants, stabilizers, plasticizers, lubricants, antioxidants, and heat stabilizers. Diisononyl phthalate was used as a plasticizer to maintain the flexibility of the shielding sheet and remove pinholes, which are micropores [28].

The application of the cast solution was performed after completely removing foreign substances through filtering and a defoaming process. The sheet was manufactured through final compression molding. The size of the sheet was 300 mm × 300 mm with a thickness of 1.15 mm ± 0.02 mm. The error range of the sheet thickness of 0.02 mm is attributed to the calendar process technology application stage during sheet manufacturing. However, the error was less than 0.05 mm, indicating that it did not affect the shielding performance [29]. To prevent pinholes from occurring during the application of the cast solution and the final molding process, the aging time of the cast solution and the compression molding temperature were adjusted. The shielding sheet fabricated through this process and the previously fabricated tungsten wire-based shielding fabric were cured at a pressure of 20 MPa for 5 min and then bonded. Significant changes were not made to the overall sheet manufacturing process, and the same pressure and process technology were applied to all to reduce the thickness error. In addition, in the process technology developed in this study, the content of tungsten powder could be increased to 90 wt% because the tensile strength of the sheet could be supplemented through fibers.

**Figure 3:**
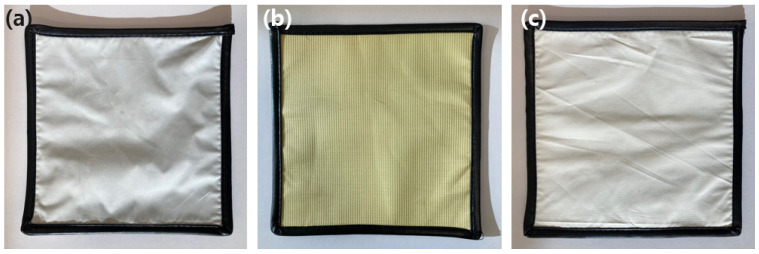
The final hybrid composite shields in which a tungsten wire-based shielding fabric is bonded to the shielding sheet surface by applying (**a**) a barium sulfate yarn, (**b**) a bismuth oxide yarn, and (**c**) barium sulfate and bismuth oxide yarns.

Figure 3 shows the appearance of the three final manufactured hybrid composite shields. Figure 3a is a shield with a barium sulfate yarn applied, Figure 3b is a shield with a bismuth oxide yarn, and Figure 3c is a shield with a barium sulfate yarn on the front side and a bismuth oxide yarn on the backside. The cross-sectional structure of the shields was confirmed using an optical microscope (OM, Axiotech 100 HD, Zeizz, Jena, Germany) and a scanning electron microscope (SEM) (JSM-5410, Jeol, Tokyo, Japan).

**Figure 4:**
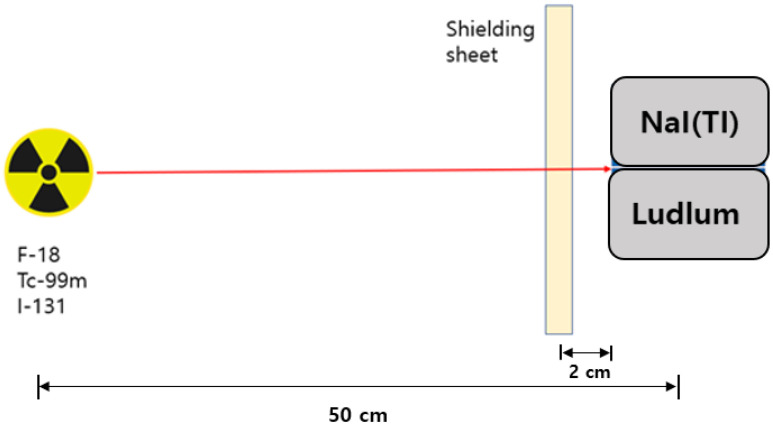
Experimental layout.

**Figure 5:**
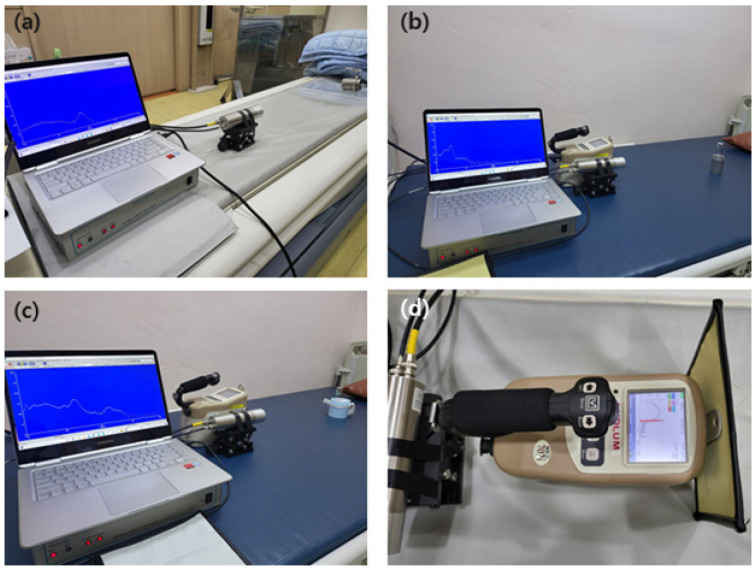
A method for testing the performance of the manufactured shields using the radioisotopes (**a**) F-18; (**b**) Tc-99m; and (**c**) I-131, measured with NaI (Harvest, Mo.6S6P1); and (**d**) measured using a Ludlum 702i.

In this study, to analyze the performance of the three shields, each experimental tool was placed in a straight line as shown in Figure 4. As shown in Figure 5, the radioactive isotopes used in the experiment were F-18 (~200 μCi), Tc-99m (~500 μCi), and I-131 (~200 μCi), and the measurement location was located at a height of 100 cm. The measuring instrument was placed at a distance of 50 cm from the source, and the developed shield was placed at a distance of 2 cm in front of the measuring instrument. The method used for the shielding test was the same as the lead equivalent test method to comply with the required guidelines (KS A 4025: 1990, 1995 confirmed) [30]. In addition, to prevent the dose from re-entering the measuring device owing to backscatter, the space was reduced to 2 cm as much as possible.

The measuring device was placed at a distance of 50 cm from the source, and the shield was installed at a distance of 2 cm from the front of the measuring device. The shielding rate was measured 10 times, and the average value was applied. Two measuring instruments were used to measure the shielding rate: NaI (Harvest, Mo.6S6P1, Birmingham, AL, USA) and Ludlum (Ludlum Measurements, Mo.702i, Sweetwater, TX, USA). Inspection and calibration of the measuring equipment was performed on May 14, 2021, and when measuring the air kerma rate, the expanded uncertainty was 3.7% for Cs-137, and the expanded uncertainty was measured to be 5.1% for the ambient dose equivalent rate. The measurement time of NaI was 120 s and that of Ludlum was 60 s [31,32].

Calculation of the measured dose was performed by determining the absorbed dose from the gamma-ray energy spectrum [33]:

(3) $$D_{\mathrm{NaI}}=\overset{n}{\underset{i=1}{\sum }}E_{i}{\left(\frac{\mu_{en}}{\rho }\right)}_{\mathrm{NaI},i}M_{i}\in \left(E_{i}\right),$$

where I is the MCA Channel, $E_{i}$ is the Channel energy, ${\left(\frac{\mu_{en}}{\rho }\right)}_{\mathrm{NaI},i}$ is the mass absorption coefficient of NaI for $E_{i}$ gamma rays, $M_{i}$ is the i-channel count, and $\in \left(E_{i}\right)$ is the detection efficiency according to the energy.

The shielding rate was calculated as in Equation (4) by substituting the case where the manufactured shield was not placed and where it was placed [34].

(4) $$\mathrm{Radiation}\;\mathrm{shielding}\;\mathrm{ratio}=1-\left(\frac{D_{\mathrm{NaI}}\;\mathrm{with}\;\mathrm{sheet}}{D_{\mathrm{NaI}}\;\mathrm{without}\;\mathrm{sheet}}\right)\times 100.$$

Additionally, the tensile strength of the shield bonded to both the bismuth oxide and barium sulfate fabrics were bonded and the shielding sheet not bonded to the shielding fabric was comparatively analyzed. An accelerated compression creep tester (INSTRON, Mo 5584, Norwood, MA, USA) was used to measure tensile strength [35].

## 3. Results

**Figure 6:**
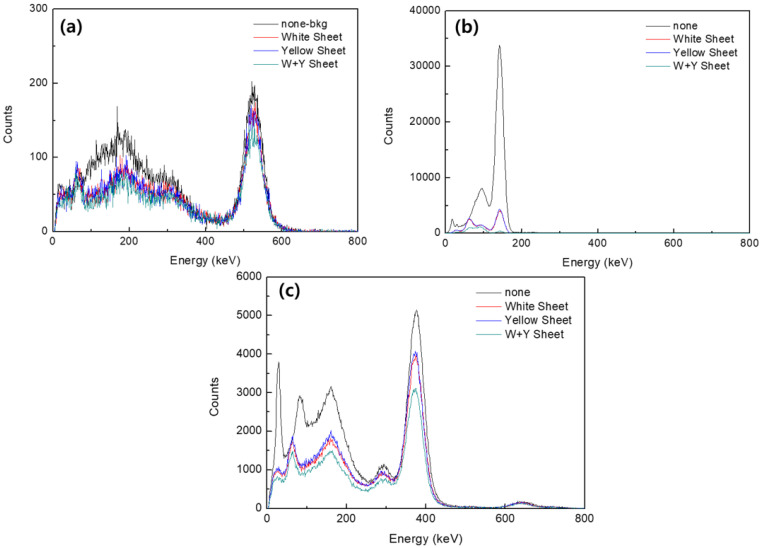
Shielding performance results by measured energy, with (**a**) the shielding performance using F-13; (**b**) the shielding performance using Tc-99m; and (**c**) the shielding performance using I-131.

The performance of the final shields according to the measured energy is shown in Figure 6. There was no obvious difference in the performance of the shields with the barium sulfate and bismuth oxide yarns applied to the tungsten wire. However, the performance was found to be improved in the shield with the barium sulfate and bismuth oxide bonded back and forth. In particular, the shielding performance measurement results according to the source Tc-99m showed excellent shielding performance.

Table 2 shows the shielding rate measurement results. As a result of measuring the shielding rate using Tc-99m as a source, the shield using the barium sulfate yarn showed an effect of improving the shielding rate by approximately 1.4% compared to the shield using the bismuth oxide yarn. In the case of the composite shield manufactured with both barium sulfate and bismuth oxide, the shielding rate against sources of F-18 and I-131 was improved by approximately 14% compared with the barium sulfate shield. In addition, a comparison of the composite shield with the bismuth oxide shield showed that the former improved the shielding rate by approximately 15.4%. Compared to the shield with no shielding fibers bonded, the composite shield exhibited a higher shielding rate of approximately 24%.

**Figure 7:**
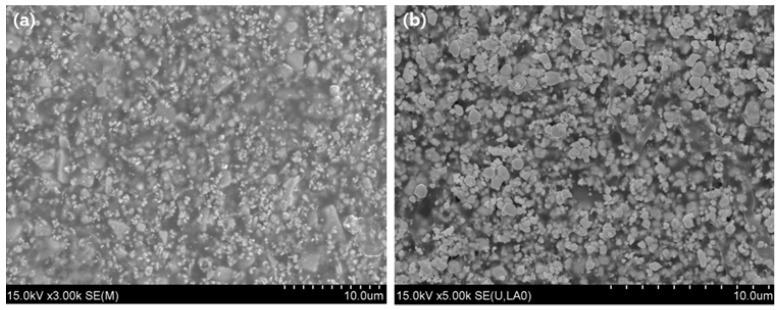
Cross-sectional SEM analysis of the fabricated sheet. (**a**) Magnification 15.0 kV × 3.00 k and (**b**) Magnification 15.0 kV × 5.00 k.

Figure 7 is a cross-sectional analysis result of the manufactured shielding sheet. As shown in Figure 7a of 15.0 kV × 3.00 k magnification and Figure 7b of 15.0 kV × 5.00 k magnification, it can be seen that tungsten particles, which are generally shielding materials, are evenly dispersed. Furthermore, the tensile strength of the composite shield was measured to be 71.6 MPa lengthwise and 52.2 MPa widthwise, while the same tensile strengths of the shielding sheet without bonding the fabric measured as only 28.2 MPa and 26.4 MPa, respectively. Based on this, it was confirmed that the tensile strength, the basic physical property of the shielding sheet, approximately doubled when the fibers were bonded.

## 4. Discussion

Radioactive isotopes utilized for diagnostic and therapeutic purposes in medical institutions decay naturally and emit radiation. The types of radiation emitted include α-rays, β-rays, gamma rays, and X-rays, and the administered radioisotope cannot be controlled like an X-ray generator because it has its characteristics [36,37]. The emitted radiation generally has high energy and penetrating power, and since the direction of emission is not determined, radiation workers and people around the patient face a high risk of exposure. Therefore, when nuclear medicine workers inject patients with isotopes used for examination or treatment, they are exposed to the radiation emitted from the patient receiving radioactive isotopes [38].

Therefore, active defense is required for both medical personnel and patients in these environments. Defense using shields is more effective than distance and time [39]. Above all, shielding clothing that allows medical personnel to physically perform medical practices uninhibited is required, meaning the shielding fabric must be flexible and thin.

The radioactive isotopes commonly used in medical institutions are F-18 (511 keV), used in positron emission tomography, Tc-99m (140 keV), which is commonly used in imaging, and I-131 (364 keV), which is widely used in treating thyroid tumors. Each of these isotopes emits radiation at a different energy based on their unique physical properties [40]. This necessitated the development of a shield fabricated from an eco-friendly, non-lead material of which the shielding performance is at least equivalent to 0.55 mmPb [41].

The hybrid composite shield fabricated in this study exhibited a lead equivalent of 0.55 mmPb and a thickness of 1.35 ± 0.02 mm, while the shielding performance and tensile strength were also evaluated. Based on this result, the hybrid composite shield can be used as a material for an Apron, which is a shielding garment used by personnel who work in medical institutions. Tungsten has the disadvantage of being less economical than lead; however, tungsten is clearly an eco-friendly material and can reduce the thickness and weight of the shield. In addition, measurement of the thickness of the shield indicated that the error range of the suggested thickness was 0.02 mm. This error range is considered to enable the realization of mass production in a range that does not affect the shielding performance.

In the case of a sheet-based shield, previous research on the mixing conditions of the shielding material and the polymer mainly focused on the difference in shielding performance according to the type of eco-friendly shielding material [42]. Fiber-based shields are mainly used as shields against scattered radiation or low-dose radiation owing to their low shielding performance. Such shields used in medical institutions are manufactured through various process technologies, such as bonding a non-woven fabric to a sheet [43]. However, there are still some challenges in the manufacturing process, such as maintaining the reproducibility of the shielding performance and securing mass production technology. In addition, research on mixed materials to improve economic feasibility is still being conducted.

This study developed a hybrid composite shield that satisfies both thickness and flexibility by bonding the shielding fiber to the sheet, differing from the conventional method of coating the shielding mixture on the non-woven fabric. In the future, research on lightweight shielding clothing with higher tungsten content and further thickness reductions to maximize the physical freedom of medical personnel is required [44]. As a limitation of this study, only tungsten was used as a single material, and it is considered that a study that can further reduce the thickness through research on eco-friendly composite materials is needed in the future.

## 5. Conclusions

Shielding suits used for radiation protection in medical institutions in which radioactive isotopes are used restrict the mobility of the medical personnel who wear these suits. Therefore, it was attempted to ensure mobility by improving the limitations of the physical properties of the shielding clothing used in medical institutions. In this study, a new process technology that can improve the shielding performance by adding a high content of shielding material was proposed. In addition, bonding the woven shielding fiber using the composite yarn impregnated with the shielding material to the tungsten wire-based yarn successfully increased the tensile strength of the shielding sheet to overcome the existing problem. The hybrid composite shield developed with process technology that can maintain the shielding performance while reducing the thickness and weight of the shielding sheet improved the shielding performance by 10% and the tensile strength by 53% compared with the existing shielding sheet. Therefore, the improved shielding suit, realized by using a new process technology that fuses shielding sheets and shielding fibers, is expected to be commercialized so that the suit can guarantee a wider range of activities for medical personnel in medical institutions in the future.

## Figures and Tables

**Table 1:** Characteristics of the yarn and fabric of the shielding sheet surface fibers.

Composite Yarn			Shielding Fiber			
Fineness (D)	Tensile Strength (g/d)	Elongation at Break (%)	Tread Count (Thread/Inch)		Weight (g/m^2^)	Thickness (mm)
			Warp	Weft		
501.1	1.45	4.2	80	60	112–121	0.20–0.21

**Table 2:** Shielding rate by measurement isotope of the manufactured shields.

Shields	Shielding Rate of Radio-Isotope (%)			Detector
	F-18	Tc-99m	I-131	
Tungsten Shielding Sheet (0.55 mmPb)	11.9	66.4	19.3	Harvest
	12.4	69.5	22.1	Ludlum
Barium Sulfate Fabric Bonding Shield (White)	13.0	76.5	20.8	Harvest
	14.8	79.0	24.0	Ludlum
Bismuth Oxide Fabric Bonding Shield (Yellow)	15.4	75.1	18.8	Harvest
	12.6	80.7	21.6	Ludlum
Composite Fabric Bonding (White + Yellow)	25.3	90.5	36.1	Harvest
	27.0	89.5	37.3	Ludlum

## Data Availability

Data is contained within the article.
